# Investigating the association between dietary patterns and glycemic control among children and adolescents with T1DM

**DOI:** 10.1515/biol-2022-0758

**Published:** 2023-12-31

**Authors:** Reema Tayyem, Sara Zakarneh, Ghadir Fakhri Al-Jayyousi

**Affiliations:** Department of Human Nutrition, College of Health Science, Qatar University, Doha, Qatar; School of Agriculture, The University of Jordan, Amman 11942, Jordan; Department of Public Health, College of Health Science, Qatar University, Doha, Qatar

**Keywords:** dietary patterns, glycemic control, children, adolescents, T1DM

## Abstract

Nutrition plays a critical role in managing diabetes, particularly in children with type 1 diabetes mellitus (T1DM). This study aimed to investigate the dietary patterns associated with glycemic control among Jordanian children and adolescents with T1DM. A total of 107 Jordanian children and adolescents with T1DM were enrolled (53 males and 54 females) in this cross-sectional study. Data were collected through face-to-face interviews using three valid and reliable questionnaires. The study revealed that only 25.7% of the participants had good glycemic control, while almost 51% had poor glycemic control. Overall, three dietary patterns were identified in this study: “High-Vegetables,” “Unhealthy,” and “High-Fruits.” The “High-Vegetables” dietary pattern showed a protective association in controlling glycated hemoglobin at the second and third tertiles (odds ratio, CI: 0.07 (0.005–0.826); 0.06 (0.005–0.741), respectively). The “High-Vegetables” dietary pattern showed a protective effect against poor glycemic control. Although the association between the “Unhealthy” and “High-Fruits” dietary patterns and poor glycemic control did not reach significance at the tertiles level, it is noteworthy that a significant *P-*trend of 0.018 and 0.012, respectively, was observed for both patterns. We encourage children and adolescents to incorporate an assortment of whole, unprocessed vegetables into their diet in appropriate amounts to help manage their glycemic control.

## Graphical abstract

**Figure:**
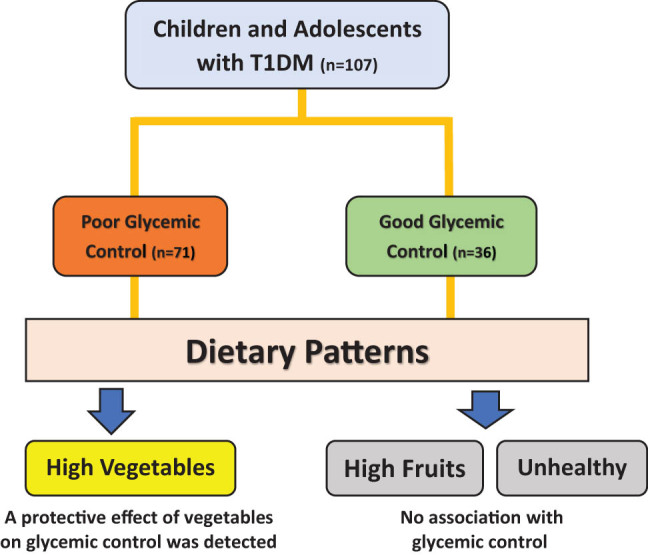

## 1 Introduction

Healthy dietary habits are not particularly common among children and adolescents with type 1 diabetes mellitus (T1DM). The diets reported by youth with T1DM often feature lower intake levels of fruits, vegetables, and whole grains than what dietary recommendations suggest. Conversely, they tend to have a higher intake of total fat and saturated fats, exceeding the recommended limits [1]. Food behaviors associated with age, such as transient food preferences, behavioral resistance, variable appetite, and food refusal, are common in this age group and are part of normal early childhood development [2].

Rather than focusing solely on individual food items, adopting food pattern approaches has revealed various dietary patterns associated with the risk of poor glycemic control [3]. Several dietary patterns that encompass combinations of different foods or food groups have proven beneficial for diabetes management [4]. These dietary patterns often include the Mediterranean diet (Med diet) and the Dietary Approaches to Stop Hypertension (DASH), both of which emphasize fruits, vegetables, whole grains, and legumes while limiting red meats, refined grains, and sugar-sweetened beverages, thereby promoting diabetes prevention. The Med diet, for instance, is characterized by a moderate energy intake, low consumption of animal fat, high intake of fruits, vegetables, whole grains, legumes, olive oil, and moderate consumption of fish, poultry, and red wine, combined with regular physical activity (25–30 min daily) [5]. Diets like the Med diet, which are based on whole grains, monounsaturated fats, and plant-based foods, and limit red and processed meats, have been shown to improve dyslipidemia, particularly low-density lipoprotein (LDL), and reduce the risk of cardiovascular diseases [6]. Numerous studies have also observed a positive association between healthy dietary intake and glycemic control.

Adherence to the DASH diet, rich in vegetables, fruits, low-fat dairy products, whole grains, poultry, fish, and nuts, while limiting saturated fat, red meat, sweets, sugar-containing beverages, and sodium, has been linked to a reduced risk of diabetes [7]. Preparing meals according to the guidelines of the Med Diet has been associated with maintaining normal daily glycemia and preventing future metabolic disorders [8]. Additionally, a high HbA1c level has been linked to a high intake of sugar-rich sweets [9], consistent with a study by Antoniotti et al. highlighting the importance of the source of carbohydrate intake and glycemic load (GL) in HbA1c levels [10]. The relationship between dietary patterns and glycemic control can be attributed to various mechanisms [11]. Factors such as nutrient composition, fiber content, antioxidants, and phytochemicals in whole foods play a role in promoting better glucose regulation [11]. Moreover, adopting a healthy dietary pattern is associated with weight management and improved insulin sensitivity, both crucial for maintaining optimal glycemic control.

According to the International Diabetes Federation (2013), the incidence of T1DM among children under 14 years old in Jordan was reported to be 3.2 per 100,000. Furthermore, a study by Alghtani et al. found an 18% increase in the prevalence of T1DM among Jordanian children between 2011 and 2016. This increase underscores a significant health concern in Jordan, highlighting the urgency of enhanced healthcare efforts [12]. The significance of this study lies in being the first in Jordan to assess the association between dietary patterns and glycemic control among Jordanian children and adolescents aged 6–18 years living with T1DM.

## 2 Materials and methods

### 2.1 Study design

A cross-sectional design was used to investigate the association between dietary patterns and glycemic control among Jordanian children and adolescents diagnosed with T1DM.

### 2.2 Sample characteristics

A convenient sampling strategy was followed to recruit participants for our study. The inclusion criteria for the study required participants with the onset of diabetes to be at least 1 year and less than 10 years, participants to be Jordanians, between 6 and 18 years of age, be on insulin, and to provide verbal and formal consent through signed consent forms by the children’s caregivers. Exclusion criteria included individuals with other chronic diseases or autoimmune diseases including cancer, food allergies, celiac disease, who are unable to communicate verbally, use insulin pumps, and newly diagnosed with T1DM. Recruitment took place at King Hussein Medical Center, specifically Al-Hussein Hospital and Queen Rania Pediatric Hospital.


**Informed consent:** Informed consent has been obtained from all individuals included in this study.
**Ethical approval:** The research related to human use has been complied with all the relevant national regulations, institutional policies and in accordance with the tenets of the Helsinki Declaration, and has been approved by the King Hussein Medical Center Institutional Review Board (IRB) committee. The IRB number for the study was 1/2019/2863.

### 2.3 Settings

Data collection for this study took place in a hospital setting, specifically at King Hussein Medical Center. The outpatient department of each hospital served as the location for data collection. Before conducting the study, the researcher obtained ethical approval from the institutional review board (IRB) of the hospital with IRB approval number 1/2019/2863. The approved proposal included the provision of a private room with suitable physical conditions for conducting interviews.

### 2.4 Data collection

During the initial meeting, the researcher explained the study’s objectives and requested participants to carefully read and sign the consent form. Parents were required to sign the consent form before data collection commenced. To ensure confidentiality, the researcher assigned each participant a unique ID and only the researcher had access to the participants’ personal information. All instruments and materials used in the study, such as questionnaires and test tubes, were labeled with the corresponding participant ID. All the used questionnaires were in Arabic.

#### 2.4.1 Instrument

Data collection for this study involved the use of a three-part package to fulfill the study objectives. The package consisted of three structured questionnaires: a Personal Information Questionnaire, a Food Frequency Questionnaire (FFQ) adapted from Tayyem et al. [13], and a Physical Activity Questionnaire adapted from Kowalski et al. [14]. The data collection method involved face-to-face interviews with the child’s caregiver, and the questionnaires were filled out by the researcher.

(a) The Personal Information Questionnaire gathered details about the parents, including age, education, marital status, occupation, family income, smoking status, and history of T1DM. Additionally, information about the child was collected, such as age, gender, school grade level, the onset of the disease, daily activities, anthropometric measurements (weight, height, body mass index [BMI]), and details about the type and doses of insulin used.
(b) Dietary assessment: Participants’ dietary data were collected using a validated Arabic FFQ, specifically designed for children and adolescents, consisting of 120 items [13]. The FFQ assessed the frequency of food consumption over the past year, with each question divided into two sections: a culturally appropriate food list and portion size information expressed in household measures or customary packing size. The food list was categorized into different sections based on food types. The frequency response options ranged from “never” to “≥6/day,” providing ten choices. To assist participants in estimating portion sizes for foods that could not be measured using standard units, food models were used. The dietary data obtained from the FFQ were analyzed using the Food Processor program (ESHA Food Processor SQL version 10.9.0.0), supplemented with additional data on locally consumed foods in Jordan. This analysis enabled the estimation of daily energy and nutrient intake. To account for foods that were not available in the Food Processor program, the study utilized food composition tables developed by Takruri et al. [15].
(c) Physical activity questionnaire: The Arabic Physical Activity Questionnaire for Older Children (PAQ-C) and Physical Activity Questionnaire for Adolescents (PAQ-A) versions were used in this study. The questionnaires are 7-day recall questionnaires designed to assess general levels of physical activity in youth from grades 4–12 (approximately ages 8–20) [14]. This questionnaire offers a reliable and valid means of evaluating physical activity with low cost and ease of administration. However, it is important to note that they do not provide detailed information on caloric expenditure, specific frequency, time, and intensity, or differentiate between different activity intensities such as moderate and vigorous activities. Instead, they provide a summary activity score to assess overall physical activity levels.

### 2.5 Anthropometric measurements

Body weight and height measurements were conducted on children, pre-adolescents, and adolescents. BMI was calculated using the formula weight (kg)/height (m^2^), following the methodology described by Lee and Nieman [16]. The BMI values were categorized into different groups based on the BMI-for-age CDC growth charts. These categories include underweight (percentile <5), healthy weight (≥5 and <85), overweight (≥85 and <95), and obese (≥95) [16]. Body weight measurements were taken using the In-Body composition analyzer (In-Body 770 for adolescents and In-Body 570 for children), with participants wearing light clothes and no shoes. Standing height was measured using a stadiometer, with the shoulder in a relaxed position and the arms hanging freely, to the nearest 0.5 cm.

### 2.6 Biochemical tests

Lab technicians drew random blood samples from only 80 participants in the overall sample to assess their biochemical indices related to nutritional status. However, 60 parents of the participants did not give consent for blood sample collection. The blood samples were analyzed for triglyceride (TG), total cholesterol, high-density lipoprotein (HDL) cholesterol, and LDL cholesterol using the enzymatic colorimetric method and Cobas (c111) enzymatic kits from Roche Diagnostics, USA. The results for HbA1c and fasting blood glucose (FBG) were obtained from the participants’ medical records.

### 2.7 Statistical analysis

All statistical analyses were conducted using SPSS version 22.0 (IBM SPSS Statistics for Windows, IBM Corporation). Descriptive analyses were conducted to examine the frequency of different variables. Chi-square was used to detect the statistical differences among categorical variables. A one-way ANOVA test was used to find the difference between continuous variables of the different age groups (children, pre-adolescents, and adolescents). Data were presented as mean ± SD. To evaluate the association between dietary patterns and HbA1c, we conducted a binary logistic regression analysis with suboptimal glycemic control (HbA1c ≥ 8.5%) [17]. Principal component analysis on the entire population was conducted using a selected set of 61 selected food items to derive dietary patterns. Factors were retained based on a fixed number of factors to extract. Then, varimax rotation was applied to the factor-loading matrix to review the correlations between variables and factors. Foods with absolute rotated factor loadings >0.30 were considered a significant contributor to the pattern. Binary logistic regression was used to calculate odds ratio (OR), CI, linear regression, and *p*-value while controlling for age, gender, type of insulin, the occurrence of disease, BMI, and energy intake (kcal) [1]. The level of significance was set at *p* < 0.05.

## 3 Results

Table 1 displays the division of patients into two groups based on their glycemic control: good glycemic control (*n* = 36) comprising 21 males and 15 females, and poor glycemic control (*n* = 71) comprising 32 males and 39 females. The distribution of variables including gender, age group, age at occurrence of disease, and physical activity score did not exhibit significant differences between the good and poor glycemic control groups (*p* > 0.05). Similarly, there were no significant differences in anthropometric measurements such as height, weight, BMI, BMI category, types of insulin, and insulin doses between the two groups. Among all the biochemical tests conducted, only TG levels showed a significant difference between the groups (*p* = 0.020).

**Table 1:** Descriptive, clinical, anthropometric, and biochemical data according to glycemic control

Variable	HbA1c ≤8.5	HbA1c >8.5	*p*-value
	*N* = (36)	*N* = (71)	
**Gender (** ***N*** **) (%)**			
Male	21 (58.3)	32 (45.1)	0.195
Female	15 (41.7)	39 (54.9)	
**Age group (** ***N*** **) (%)**			
Children	6 (16.7)	16 (22.5)	0.697
Pre-adolescents	19 (52.8)	32 (45.1)	
Adolescents	11 (30.6)	23 (32.4)	
**Mean ± SD**			
Age (years)	11.9 ± 3.2	12.0 ± 3.4	0.672
Onset of diseases (years)	3.3 ± 2.3	4.0 ± 2.7	0.168
Physical activity level	2.1 ± 0.55	2.1 ± 0.50	0.285
Weight (kg)	42.7 ± 16.4	46.7 ± 19.2	0.488
Height (cm)	146.5 ± 17.8	144.6 ± 23.0	0.415
BMI (kg/m^2^)	19.3 ± 3.9	20.5 ± 4.1	0.533
**BMI category (** ***N*** **) (%)**			
Underweight	4 (11.1)	2 (2.8)	0.095
Normal weight	21 (58.3)	43 (60.6)	
Overweight	9 (25)	13 (18.3)	
Obese	2 (5.6)	13 (18.3)	
**Types of insulin (** ***N*** **) (%)**			
Rapid-acting	2 (5.6)	5 (7)	0.070
Pre-mixed	16 (44.4)	45 (63.4)	
Long-acting	2 (5.6)	0	
Rapid + long	16 (44.4)	21 (29.6)	
**Doses of insulin (mean ± SD)**			
Morning	1.3 ± 4	0.8 ± 3.1	0.233
At breakfast	9.5 ± 5.9	13.2 ± 6.9	0.144
At lunch	10.8 ± 5.8	14.3 ± 7.3	0.113
At dinner	7.6 ± 5.5	9.9 ± 6.1	0.770
At bedtime	7.1 ± 10.5	5.3 ± 9.5	0.216
Total of insulin	36.3 ± 19.0	43.5 ± 21.0	0.493
**Biochemical tests (mean ± SD)**			
HbA1c (*n* = 107)	7.6 ± 0.8	10.9 ± 1.9	<0.001
FBG (*n* = 107)	148.8 ± 66.2	174.3 ± 97.4	0.288
Cholesterol (*n* = 80)	166.2 ± 37.8	168.2 ± 33.7	0.444
LDL (*n* = 80)	95.3 ± 32.3	92.0 ± 30.6	0.627
HDL (*n* = 80)	55.8 ± 16.2	54.9 ± 15.4	0.996
TG (*n* = 80)	103.0 ± 46.5	131.6 ± 113.6	0.020

Values are mean ± SD or *n* (%), and significance is at *p* ≤ 0.05.

Underweight = BMI% <5; healthy weight ≥5 and <85; overweight ≥85 and <95; obese ≥95. Abbreviations: BMI: body mass index; FBG: fasting blood glucose; LDL: low-density lipoprotein; HDL: high-density lipoprotein; TG: triglycerides.

To identify dietary patterns, we calculated the factor-loading matrix scores, which illustrate the correlations between food groups and dietary patterns. The results are presented in Table 2. Through factor analysis, three dietary habits were identified and named based on the food groups with the highest positive loadings: “High-Vegetable,” “Unhealthy,” and “High-Fruits” patterns. The “High-Vegetable” pattern had the highest loading in cooked vegetables, mixed vegetable salad, fresh tomato, cucumber, garden rocket, peas, and onion. The “Unhealthy” pattern was characterized by high loadings of candy, biscuits, soft drinks, juices, and chocolates. The “High-Fruits” pattern was associated with dried fruit, pears, mango, grapefruits, and grapes. These dietary patterns explained a total variance of 37.6%, with 16.3% attributed to the “High-Vegetable” pattern, 12.2% to the “Unhealthy” pattern, and 9.1% to the “High-Fruits” pattern. A Kaiser–Meyer–Olkin (KMO) test and Bartlett’s test of sphericity were used to assess suitability for using factor analysis for this exercise. Sampling adequacy and inter-correlation of factors were supported by a KMO value >0.673 and Bartlett’s test of sphericity <0.001, respectively. Factors were retained based on an eigenvalue of >1.25 for the screen plot.

**Table 2:** Factor loading matrix for the three major dietary patterns identified in a representative sample

Food group	Dietary patterns		
	High-vegetables	Unhealthy	High-fruits
Vegetable salad	0.828		
Tomato	0.760		
Parsley	0.747		
Labaneh	0.695		
Yogurt	0.691		
Cooked vegetables	0.680		
Cucumber	0.661		
Garden rocket	0.644		
Minced meat	0.612		0.350
Cooked leafy vegetables	0.577	−0.312	0.404
Rice	0.550		
Apple	0.542		
Onion	0.539		
Egg	0.537		
White cheese	0.509		
Chicken	0.508		
Lettuce	0.494		
Sweet pepper	0.478		0.391
Green beans	0.433	−0.310	
Peas	0.432		
Olive	0.430		0.340
Spared cheese	0.410		
Buttermilk	0.384		
Cauliflower	0.373		
Carrot	0.345		
Pickles	0.323		
Candy		0.770	
Chocolate		0.682	
Melon		0.665	0.440
Biscuits filled with cream		0.652	
Salty biscuits		0.595	
Cake		0.584	
Soft drinks		0.563	
Hummus tahini		0.560	
Biscuit		0.559	0.394
White bread		0.533	
Ice cream		0.523	
Strawberries		0.510	
Falafel		0.505	
Potato chips		0.483	
Dessert		0.471	
Popcorn		0.427	
Ma’moul	−0.339	0.385	0.310
Hotdog		0.360	
Banana		0.354	0.334
Artificial <30% juice concentrate		0.354	
Turkey		0.351	
Pears			0.706
Dried fruits			0.700
Mango			0.697
Oat			0.638
Grapefruits			0.597
Grapes			0.584
Watermelon		0.523	0.570
Broccoli	0.303		0.566
Kaki			0.538
Macaroni salad			0.527
Pineapple			0.503
Bulger			0.494
Peach	0.481		0.473
Coleslaw			0.377
A variance of Intake Explained%	16.3%	12.2%	9.1%

The good glycemic control group was considered the reference group for dietary pattern analysis.

ORs and the corresponding 95% CI of the good and poor glycemic control groups by tertiles of factor scores and continuous factor scores for the three dietary patterns are shown in Table 3. Results refer to the composite model including all the dietary patterns simultaneously, together with the relevant confounding variables. A significant protective association of controlling HbA1c was detected with the “High-Vegetable” pattern at the second and third tertiles (OR, CI: 0.07 [0.005–0.826]; 0.06 [0.005–0.741], respectively). In contrast, no significant association was found in the “Unhealthy” pattern or “High-Fruits” in both tertiles (OR, CI: 1.31 [0.279–6.116] T2; 1.93 [0.125–6.84] T3; and 2.68 [0.551–13.049] T2, 2.42 [0.378–15.510] T3, respectively).

**Table 3:** Association between glycemic control and dietary patterns among the study samples

Dietary patterns	T1	T2 OR (95% CI)	T3 OR (95% CI)	*P*-trend
High-vegetable	1	0.07 (0.005–0.826)	0.06 (0.005–0.741)	0.007
Unhealthy	1	1.31 (0.279–6.116)	1.93 (0.125–6.84)	0.018
High-fruits	1	2.68 (0.551–13.049)	2.42 (0.378–15.510)	0.012

OR and CI: odd ratio and confidence interval, and OR was adjusted for age, gender, type of insulin, the occurrence of disease, BMI, and energy (kcal). –The good glycemic group was considered the reference group for dietary pattern analysis.

## 4 Discussion

In this cross-sectional study involving children, pre-adolescents, and adolescents with T1DM (*n* = 107), we examined the dietary patterns associated with glycemic control. We identified three distinct dietary habits: the “High-Vegetable” pattern characterized by high consumption of cooked vegetables, mixed vegetable salad, fresh tomato, cucumber, Rocca, peas, and onion; the “Unhealthy” pattern characterized by high consumption of candy, biscuits, soft drinks, juices, and chocolates; and the “High-Fruit” pattern characterized by high consumption of dried fruits, pears, mango, grapefruits, and grapes, with minced meat consumption. Our findings revealed that the “High-Vegetable” pattern was significantly associated with better glycemic control in the second and third tertiles (OR, CI: 0.07 [0.005–0.826]; 0.06 [0.005–0.741]), respectively. Although studies specifically examining the association between high vegetable consumption and glycemic control in T1DM are limited, Albadri et al. and Øverby et al. found that adolescents with optimal glycemic control had a higher intake of vegetables and fruits compared to those with suboptimal control [18,19]. Additionally, Yen et al. demonstrated that increased raw vegetable intake led to improved glycemic control in adults with T2DM after a 12-week intervention [20]. Basu et al. conducted a study investigating the relationships between dietary food groups and HbA1c levels at baseline and after 6 years [21]. Their analysis, adjusted for factors like age, gender, BMI, total calorie intake, and diabetes status, revealed that no significant associations were found in the fruit and vegetable groups, except for two noteworthy findings. Dark green vegetables were inversely associated with baseline HbA1c levels, while tomatoes were inversely associated with HbA1c levels after 6 years [21]. On the contrary, a food group described as “baked desserts,” including items like commercial and homemade cookies, candies, cakes, and pies, showed a significant positive association with HbA1c levels at both time points [21]. It is worth noting, however, that certain prospective cohort studies have suggested that dietary fibers from vegetables may not significantly reduce the risk of diabetes, as documented by Weickert and Pfeiffer [22].

Another study conducted by Dominguez-Riscart et al. reported an improved HbA1c in the group with optimal adherence to the Med diet [23]. The traditional Med diet is characterized by high consumption of vegetables, fruits and nuts, legumes, and unprocessed cereals; low consumption of meat and meat products; and low consumption of dairy products. The authors attributed this improvement to the lower energy and calories in vegetables as well as higher fiber content [23]. Similarly, the Li et al. findings indicated that vegetable intake, but not fruit intake, had an inverse association with fasting plasma glucose levels in patients with diabetes and kidney transplants [24]. This relationship was dose-dependent, meaning that as vegetable intake increased, fasting plasma glucose decreased. Specifically, each 100 g increase in vegetable intake was associated with an 11.6% reduction in fasting plasma glucose [24]. Lodefalk and Åman found that patients with better glycemic control tended to focus on consuming more fruits and vegetables and consuming fewer simple sugars compared to those with poorer glycemic control [25].

In our study, we did not find a significant association between the “Unhealthy” and “High-Fruits” dietary patterns and glycemic control in both tertiles (OR, CI: 1.31 [0.279–6.116] T2; 1.93 [0.125–6.84] T3; and 2.68 [0.551–13.049] T2, 2.42 [0.378–15.510] T3, respectively). However, the *P-*trend showed a significant association between “Unhealthy” and “High-Fruits” dietary patterns and glycemic control. This finding aligns with another study, which highlighted that aside from the overall daily carbohydrate intake, the origin of carbohydrate consumption, and consequently, the GL, are correlated with HbA1c levels. Zakarneh et al. (2024) highlighted the connection between inadequate diabetic control and the consumption of unhealthy food items, particularly noting that a high HbA1c level was predominantly attributed to the consumption of sugary sweets [26]. Additionally, the authors demonstrated that the total daily carbohydrate intake, the source of carbohydrate intake, and hence, GL can all affect HbA1c levels [25]. Xu et al. reported a significant association between consuming higher total fat and higher HbA1c concentrations, while higher saturated fat, protein, and sucrose intakes were marginally associated with higher HbA1c concentrations [27]. Additionally, in contrast, a randomized controlled trial examined the impact of a fruit-rich diet compared to a low-fruit diet on liver steatosis, lipid profile, and glycemic control in individuals with non-alcoholic fatty liver disease. Following a 6-month intervention, the group on the fruit-rich diet exhibited a significant increase in dyslipidemia (*p* < 0.001), FBG (*p* < 0.001), and insulin resistance (*p* < 0.001) compared to the control group. Conversely, the control group demonstrated improvements in lipid profile (*p* < 0.05) and insulin resistance (*p* < 0.001) [28]. Additionally, Bazzano et al. found that increasing fruit consumption to three servings per day was associated with a reduced risk of diabetes (OR = 0.82 [CI: 0.72–0.94]), while a similar increase in fruit juice intake was linked to an increased risk of diabetes (OR = 1.18 [CI: 1.10–1.26]) [29].

## 5 Study limitations and strengths

The study has certain limitations due to the nature of the sample and the methodology employed. The reliance on participants’ ability to recall accurate information regarding food consumption and physical activity may introduce recall bias. Although the study results are aligned with the findings of many well-conducted studies, we cannot generalize the results to all the pediatrics in Jordan due to the small sample size. Additionally, some children may have been hesitant to report certain types of sweets consumed due to parental influence. Furthermore, the study did not account for the potential impact of cooking on nutrient bioavailability. However, the study also has notable strengths. The use of a validated and detailed FFQ allowed for comprehensive dietary data collection. The FFQ demonstrated excellent reproducibility and good relative validity for most food groups in Jordanian children and adolescents. Moreover, this study represents the first attempt to assess the dietary patterns and lifestyle factors specifically among children and adolescents with T1DM.

## 6 Conclusion

Interestingly, the study found a protective effect of the “High-Vegetables” dietary pattern against poor glycemic control, while no significant association between the “Unhealthy” and “High-Fruits” dietary patterns and glycemic control in the second and third tertiles has been detected. However, the values of the *P-*trend showed a likelihood of significant risk for having poor glycemic control in both “Unhealthy” and “High-Fruits” dietary patterns. The findings of the study revealed that participants with poor glycemic control showed higher levels of TG compared to those with good glycemic control. Based on our study results, we actively advocate and motivate children and adolescents to include a variety of whole, unprocessed vegetables in their diet, in suitable quantities, to aid in the management of their glycemic control. However, further research utilizing prospective studies is required to validate our findings.
