# Erratum to “Investigating the association between dietary patterns and glycemic control among children and adolescents with T1DM”

**DOI:** 10.1515/biol-2022-0300

**Published:** 2024-01-30

**Authors:** Reema Tayyem, Sara Zakarneh, Ghadir Fakhri Al-Jayyousi

**Affiliations:** Department of Human Nutrition, College of Health Science, Qatar University, Doha, Qatar; School of Agriculture, The University of Jordan, Amman 11942, Jordan; Department of Public Health, College of Health Science, Qatar University, Doha, Qatar

In the published article “Tayyem R, Zakarneh S, and Al-Jayyousi GF. Investigating the association between dietary patterns and glycemic control among children and adolescents with T1DM. Open Life Sci. 2023;18(1):20220758. doi: 10.1515/biol-2022-0758”, the authors mistakenly did not include Funding information. The authors admit to the error and apologize to the editor, the staff of the journal and the journal readers for the mistake and any inconvenience it caused. The Funding information should be as follows:

**Funding information:** The open-access funding for this manuscript has been provided by the Qatar National Library.

